# Prognostic Relevance of Altered Lymphocyte Subpopulations in Critical Illness and Sepsis

**DOI:** 10.3390/jcm8030353

**Published:** 2019-03-12

**Authors:** Philipp Hohlstein, Hendrik Gussen, Matthias Bartneck, Klaudia Theresa Warzecha, Christoph Roderburg, Lukas Buendgens, Christian Trautwein, Alexander Koch, Frank Tacke

**Affiliations:** 1 Department of Medicine III, RWTH-University Hospital Aachen, 52074 Aachen, Germany; phohlstein@ukaachen.de (P.H.); hgussen@ukaachen.de (H.G.); matthiasbartneck@googlemail.com (M.B.); klaudia.kaczmarski@rwth-aachen.de (K.T.W.); croderburg@ukaachen.de (C.R.); lbuendgens@ukaachen.de (L.B.); ctrautwein@ukaachen.de (C.T.); akoch@ukaachen.de (A.K.); 2 Department of Gastroenterology/Hepatology, Charité University Medical Center Berlin, 13353 Berlin, Germany

**Keywords:** ICU, adaptive immunity, mortality, prognosis, flow cytometry

## Abstract

Lymphopenia and functional defects in lymphocytes may impact the prognosis in patients with critical illness or sepsis. Therefore, we prospectively analyzed peripheral blood leukocytes from 63 healthy volunteers, 50 non-critically ill standard care (SC) patients with infections, and 105 intensive care unit (ICU) patients (52 with sepsis, 53 without sepsis) using flow cytometry. Compared to healthy volunteers, SC and ICU patients showed significant leukocytosis, especially in sepsis, while lymphocyte numbers were significantly decreased. All major lymphocyte populations (B, T, and natural killer (NK) cells) decreased in ICU patients. However, we observed a relative reduction of T cells, alongside decreased CD8+ T cells, in critically ill patients, independent of sepsis. High absolute T cell counts (>0.36/nL) at ICU admission were associated with a significantly reduced mortality, independent of patient’s age. Moreover, patients that survived ICU treatment showed dynamic changes within 48 h towards restoration of lymphopenia and T cell depletion, while non-surviving patients failed to restore lymphocyte counts. In conclusion, the flow-cytometric analysis of peripheral blood revealed striking changes in circulating lymphocyte subsets in critically ill patients, independent of sepsis. Lymphopenia and T cell depletion at ICU admission were associated with increased mortality, supporting their relevance as predictive biomarkers and potential therapeutic targets in intensive care medicine.

## 1. Introduction

Inappropriate systemic inflammation is a key characteristic in critically ill medical patients admitted to the intensive care unit (ICU) [1], especially in patients with sepsis [2]. In fact, sepsis is nowadays defined as a dysregulated host response to an infection causing a life-threatening organ dysfunction [3]. Interestingly, patients with sepsis typically display defects in innate as well as adaptive immunity [4]. While leukocytes and systemic cytokines are highly prevalent during the onset of sepsis [5], granulocytes have an immature phenotype that suppresses adaptive immunity (oftentimes termed “myeloid-derived suppressor cells”) [5]. Because of this immune-suppressive myeloid phenotype, lymphocytes likely upregulate markers of cell exhaustion [6] and are unable to mount proper cytokine responses [7]. Altogether, these features of immunosuppression have been convincingly linked to the risk of sepsis and organ failure in patients with infections [8] as well as to increased mortality due to sepsis [7]. The assessment of the granular characteristics of immune cells from peripheral blood leukocytes by flow cytometry has been proposed and validated in multicentric analyses to identify patients at risk for deterioration [8,9]. Moreover, the in-depth understanding of immune pathogenesis may offer novel, tailored therapeutic interventions in intensive care medicine [10].

Lymphocytes have long been recognized as a key component of dysregulated immune responses in critical illness and particularly in sepsis [6,11]. An effective functioning pool of lymphocytes is essential to control and then eradicate infections [4]. For instance, CD4^+^ T cells are activated in response to antigen presentation by dendritic cells or monocytes, release immune-regulatory cytokines, and orchestrate cytotoxic CD8+ T cell functions [4]. Natural killer (NK) cell subsets contribute to cytotoxic and cytokine-releasing activities [12], and B cells support humoral immune responses [4]. At the onset of sepsis, lymphocyte numbers are typically low [6]. During the acute phase of sepsis (i.e., the first seven days in patients), lymphocytes—T cells in particular—upregulate the inhibitory receptors cytotoxic T lymphocyte antigen-4 (CTLA4), T cell immunoglobulin mucin-3 (TIM-3), lymphocyte activation gene 3 (LAG-3), or interleukin-7 (IL-7) receptor [6]. Recovery from these immune cell alterations is generally associated with a favorable outcome [4].

Many of the mentioned studies have used sophisticated staining panels for leukocyte subset activation markers and/or ex vivo stimulation of lymphocytes to assess, for instance, cytokine release by T cells. While these studies have generated insightful data on the pathogenic involvement of leukocyte subsets in the course of sepsis, they are technically demanding, which limits their practicability in clinical routine. In addition, most experimental data were generated in patients with either infections or sepsis, while the role of leukocytes and, particularly, of lymphocyte subsets in non-septic critical illness is less clear. In our prospective, mono-centered cohort study, we therefore addressed the following aims: (a) assess the composition and clinical relevance of leukocytes and lymphocyte subsets using a straightforward flow-cytometric subset characterization in critically ill patients with and without sepsis, and (b) test whether lymphocyte subsets obtained at ICU admission and 48 h later hold prognostic value in critically ill medical patients in general.

## 2. Experimental Section

### 2.1. Patients and Controls

This study was approved by the local ethics committee (EK 150/06) of the University Hospital Aachen, RWTH Aachen, and written informed consent was obtained from every participant or authorized relatives in case of loss of consciousness. Critically ill patients were prospectively included between October 2013 and March 2015 from the intensive care unit and standard care wards of the Department of Medicine III of the RWTH University Hospital in Aachen, following an established protocol [13,14]. Septic ICU patients had a clinically suspected or verified infection diagnosed by the intensive care physicians and were subsequently treated with antibiotics. Sepsis was established following a diagnosed infection and an increase in the Sepsis-related Organ Failure Assessment (SOFA) score greater than or equal to two points [3]. Non-critically ill patients, admitted because of infectious diseases to the standard care ward, served as a diseased control population. Those patients were admitted to the hospital following a diagnosis of infection by the treating physician (based on clinical judgment, laboratory results, and/or microbial cultures) and received antibiotic therapy. Healthy volunteers from the local blood transfusion institute served as a healthy control population. Blood samples of the recruited patients were obtained by peripheral venipuncture or from inlying central venous or arterial catheters at admission and 48 h after admission. Adding 250 units of heparin (Rotexmedica, Frittach, Germany) per milliliter blood prevented coagulation of the blood samples.

### 2.2. Isolation of Peripheral Blood Mononuclear Cells

Peripheral blood mononuclear cells (PBMC) were isolated by using a Ficoll-based density gradient. Blood and cells were kept at 4 °C during the whole procedure to ensure minimal cell activation. Whole blood was mixed with an equal amount of phosphate-buffered saline (PBS, PAN Biotech Aidenbach, Germany), then carefully manually layered over 1077 Lymphocyte Separation Medium (PAA, Pasching, Austria), followed by a centrifugation at 1600 rpm for 40 min without the use of a brake at room temperature. The intermediate layer containing the PBMC was then carefully harvested, washed with PBS, and centrifuged at 1300 rpm for 10 min three times. Subsequently, the cells were resuspended in PBS and counted using a Neubauer chamber for antibody staining.

### 2.3. Flow Cytometry

Two million cells were resuspended in PBS and blocking buffer (2% bovine serum albumin, 2% rabbit serum, 2% human serum, 2% mouse serum, 2% rat serum) to block unspecific binding of antibodies. PBMC were stained with fluorescence-conjugated antibodies (CD14, CD56, CD45, CD3, CD4, CD19 by eBioscience; CD16, CD8 by BD Pharmingen). After 30 min of light-protected incubation, the cells were washed, centrifuged, and subjected to flow-cytometric analysis using a FACS Canto-II (BD, Heidelberg, Germany). In a subsequent analysis using FlowJo software (TreeStar Inc., Ashland, OR, USA), cell populations were defined after exclusion of doublets by antibody positivity in the following manner: T cells as CD3^+^CD56^-^ (subsequent analysis for CD4 and CD8), natural killer cells as CD3^-^CD19^-^CD56^+^ (subsequent analysis of CD16 and CD56), and B cells as CD19^+^. Absolute cell numbers were calculated on the basis of automated differential white blood cell counts. 

### 2.4. Statistical Analysis

Data were analyzed using SPSS (version 22, IBM Corp., Armonk, NY, USA) and GraphPad Prism 5 (GraphPad Software, Inc., La Jolla, CA, USA). As a normal distribution of samples could not be assumed, the Kruskal–Wallis test followed by a post hoc testing by Dunn’s multiple comparison test was used for more than two groups, the two-tailed Mann–Whitney U test was used for two groups of unpaired samples, and the two-tailed Wilcoxon signed rank test was used for paired samples. A significance level of α = 0.05 was used in all corresponding calculations. Associations with survival were assessed by multivariate Cox regression, and patient survival was depicted by Kaplan–Meier curves using SPSS. The Youden index was calculated to identify the optimal cut-off values for parameters to discriminate prognosis [15]. Correlations of lymphocyte subpopulations to clinical or laboratory parameters and to age as a potential confounding factor were analyzed by Spearman’s rank correlation test. To control for age as a confounding variable, all analyses comparing different subgroups were also performed with age-matched study populations.

## 3. Results

### 3.1. Peripheral Blood Leukocytosis and Lymphopenia are Characteristics of Critical Illness

**Figure 1:**
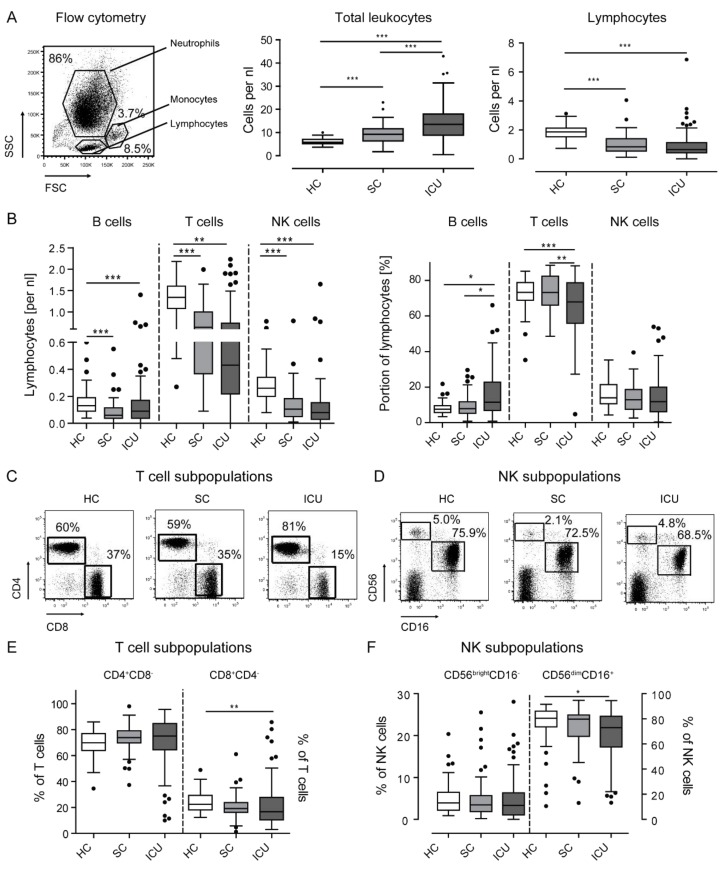
Leukocytes and lymphocyte subsets in healthy volunteers, standard care patients with infection, and intensive care patients. Leukocytes were harvested from peripheral blood using density-gradient centrifugation and subjected to flow cytometry. (**a**) A representative forward vs sideward scatter plot of the different cell types from whole blood is shown. The distributions of leukocytes and lymphocytes in the different patient cohorts are shown as box plots. Dots indicate outliers. (**b**) Circulating B, T, and NK cells are depicted by box plots as absolute cells counts and as percentage of all lymphocytes. (**c**,**d**) Representative FACS plots to identify CD4- and CD8-positive T cells (**c**) pregated on CD3^+^CD56^-^ lymphocytes as well as CD56- and CD16-expressing NK cells (**d**) pregated on CD3^-^CD56^+^ lymphocytes. (**e**) Percentages of CD4- and CD8-positive T cells. (**f**) Percentages of CD56^bright^CD16^-^ and CD56^dim^CD16^+^ NK cells. Statistics: * indicates *p* < 0.05, ** *p* < 0.01, *** *p* < 0.001. For comparison of two groups, the Mann–Whitney U test was used, for more than two groups the Kruskal–Wallis test was performed followed by a post hoc test by Dunn´s multiple comparison test. Sample sizes: Healthy controls (HC) *n* = 63, standard care (SC) *n* = 50, intensive care unit (ICU) *n* = 105.

The numbers and composition of circulating leukocyte subsets might be related to the severity and the clinical course of critical illness [9]. We therefore prospectively enrolled 63 healthy controls (HC), 50 standard care (SC) patients with ongoing infection diagnosed less than 24 h before, and 105 intensive care unit (ICU) patients submitted to the ICU less than 24 h before (Table 1), in order to conduct a detailed flow-cytometric analysis of circulating leukocytes. Among the ICU patients, *n* = 52 (49.5%) had been admitted because of sepsis. Compared to healthy volunteers, patients in SC or ICU wards showed a significant increase in leukocytes (Figure 1A), which was expectedly pronounced in critically ill patients with sepsis (Table 1). On the contrary, the absolute numbers of lymphocytes were significantly decreased in infected SC and in ICU patients (Figure 1A).

We next focused on the different lymphocyte populations in peripheral blood and determined the composition of lymphocyte subsets both as absolute numbers (per nL blood) and by their relative contribution to the circulating lymphocytes (Figure 1B). NK as well as B cells declined by absolute numbers in SC and ICU patients compared with HC, and T cells were even reduced in the lymphocyte pool. On the contrary, B cell numbers decreased in SC and ICU patients as well but constituted a larger proportion of lymphocytes in critically ill patients (Figure 1B). For further analysis, subpopulations of T and NK cells were studied in order to assess CD4- and CD8-positive T cells as % of T cells (Figure 1C), and CD56^bright^CD16^-^ versus CD56^dim^CD16^+^ NK cell subpopulations, respectively (Figure 1D). In ICU patients, CD8^+^ T cells were particularly reduced, while CD4^+^ T cells accounted for the vast majority of circulating T cells in all cohorts (Figure 1E). Regarding NK cell subsets, CD56^dim^CD16^+^ NK cells were moderately reduced in ICU patients, while CD56^bright^CD16^-^ cells remained unchanged (Figure 1F).

To analyze the possibility of age as a confounder in the determination of the different leukocyte populations, we performed a correlation analysis (Spearman’s rho correlation test), which revealed an inverse correlation between age and leukocytes and positive correlations between age and lymphocytes, B, T, and NK cells over the whole study population (i.e., control populations and ICU patients combined, data not shown). To control for age as a potentially confounding variable, we generated age-matched populations (HC *n* = 33, SC *n* = 33, ICU *n* = 81; median age 58.5 years), maintaining the original case–control distribution. The same analyses as seen in Figure 1 were performed on an age-adjusted study cohort with mean age of 58 to 59 (data not shown). Age did not differ across the patient cohorts (*p* = 0.518) in these age-adjusted cohorts. The significant differences depicted in Figure 1 were overall retained in age-adjusted subgroups, except for the difference in B cells per nL between HC and ICU patients (which were not significant anymore).

### 3.2. Lymphopenia in Critical Illness is Independent of Sepsis

**Figure 2:**
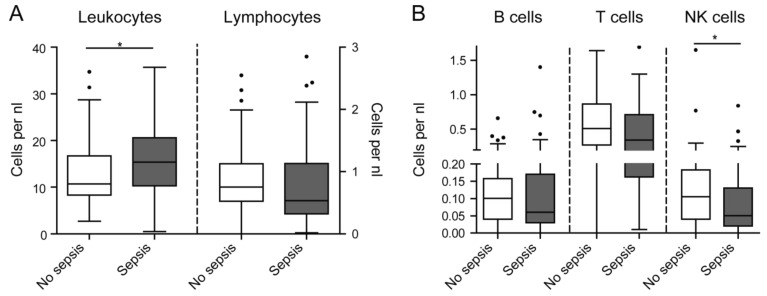
Leukocyte subsets in ICU patients with and without sepsis. (**a**) Absolute numbers of circulating leukocytes and lymphocytes in septic and non-septic ICU patients. (**b**) Lymphocyte subset distribution in the respective cohorts. Statistics: * indicates *p* < 0.05. For comparison of two groups, the Mann–Whitney U test was used. Sample sizes: No sepsis *n* = 53, sepsis *n* = 52.

Immunosuppressive features of circulating leukocytes, such as lymphopenia, have been particularly related to sepsis [7,8]. We therefore compared the subgroups of ICU patients admitted because of non-sepsis or sepsis, on the basis of current definitions [3]. While leukocytes (especially neutrophils) were significantly higher in ICU patients with sepsis, absolute lymphocyte numbers did not differ between critically ill patients with or without sepsis (Figure 2A). Regarding lymphocyte subpopulations, only NK cells were significantly less frequent in septic as compared to non-septic patients (Figure 2B). Age did not differ when comparing septic to non-septic patients (*p* = 0.118), thereby excluding it as a confounder in this analysis. The statistical analysis of CD4- and CD8-positive T lymphocytes as well as CD56^bright^CD16^-^ and CD56^dim^CD16^+^ NK cells did not reveal significant differences between septic and non-septic ICU patients (data not shown). However, flow-cytometric assessment of leukocyte subsets revealed some weak correlations between cell populations and inflammatory biomarkers. Total lymphocytes, but also T cells, inversely correlated with interleukin-10 (IL-10, Table 2). Furthermore, NK cells inversely correlated with procalcitonin (PCT, Table 2). All other significant correlations were found to be negligible (Spearman’s rho below ±0.3).

### 3.3. Lymphopenia and T Cell Depletion are Associated with Mortality in ICU Patients

**Figure 3:**
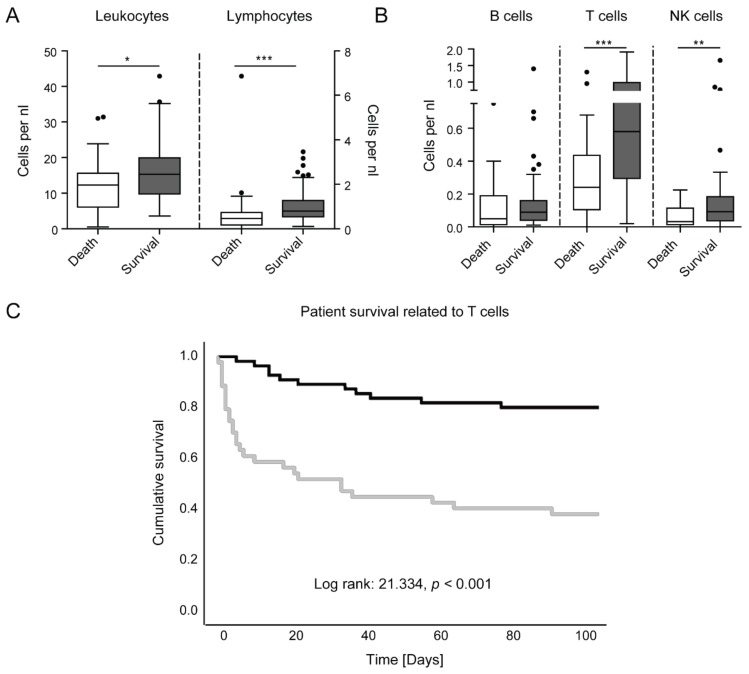
Prognostic value of lymphocyte subsets in ICU patients. (**a**) Leukocytes and lymphocytes, as obtained at ICU admission, in patients who subsequently died or survived in the ICU. (**b**) Lymphocytes subset distribution at ICU admission in patients surviving or dying in the ICU. (**c**) Kaplan–Meier curve for T cells lower than or equal to 0.36 per nanoliter (grey) and higher than 0.36 per nanoliter (black) in ICU patients. Censored events are indicated by a crossing vertical line. Statistics: * indicates *p* < 0.05, ** *p* < 0.01, *** *p* < 0.001. For comparison of two groups, the Mann–Whitney U test was used. Cox regression was performed to determine the prognostic value. Cutoff values of the Kaplan–Meier curve were determined by the Youden index. Sample sizes: death *n* = 35, survival *n* = 70.

Lymphopenia has been related to adverse outcome in sepsis, and this has been functionally linked to the exhaustion of T cells [6,7]. We therefore analyzed the possible link between circulating leukocyte subsets and mortality in critically ill patients (Figure 3). Critically ill patients that survived the ICU treatment showed significantly higher numbers of leukocytes and lymphocytes at ICU admission (Figure 3A). In particular, T and NK cells were significantly higher in surviving ICU patients compared to patients succumbed to death (Figure 3B). In a subgroup analysis comparing septic to non-septic patients, those changes were retained (detailed data not shown). In addition, surviving sepsis patients had significantly higher levels of B cells per nL (*p* = 0.009), while surviving non-septic patients showed significantly higher percentages of CD8^+^ T cells (*p* = 0.037). To control for age as a confounding variable (which did differ between surviving and non-surviving patients, *p* = 0.048), we performed the same analyses on the age-adjusted study population (in which age did not differ between surviving and non-surviving patients, *p* = 0.293). Here, the changes, as seen in Figure 3A,B, were retained, except for NK cells.

For a more granular analysis, we calculated Cox regressions for all cell types (omnibus test of model coefficients, leukocytes *p* = 0.246, lymphocytes *p* = 0.086, B cells *p* = 0.455, T cells *p* = 0.008, and NK cells *p* = 0.641 for variables in the equation). Only T cells showed a statistical significance with respect to survival. In a multivariate cox regression, age alone yielded for a hazard ratio (HR) of 1.038 (95% confidence interval 1.015–1.061, *p* = 0.001), thus age was not a significant predictor of mortality in our ICU cohort. Adding T cells per nL to the model accounted for a significant difference to the previous model (*p* = 0.002), with a HR of 0.319 for T cells (95% confidence interval 0.137–0.744, *p* = 0.008), describing T cells as a beneficial factor for patient survival (data not shown). Using the Youden index, we calculated an optimal cutoff value of 0.36 T cells per nL. By Kaplan–Meier curve analysis (Figure 3C), the chances for survival of ICU patients with more than 0.36 T cells per nL were almost doubled compared to ICU patients with low T cell numbers.

### 3.4. Lymphopenia and T Cell Depletion are Rapidly Restored in Surviving Compared to Non-Surviving ICU Patients

**Figure 4:**
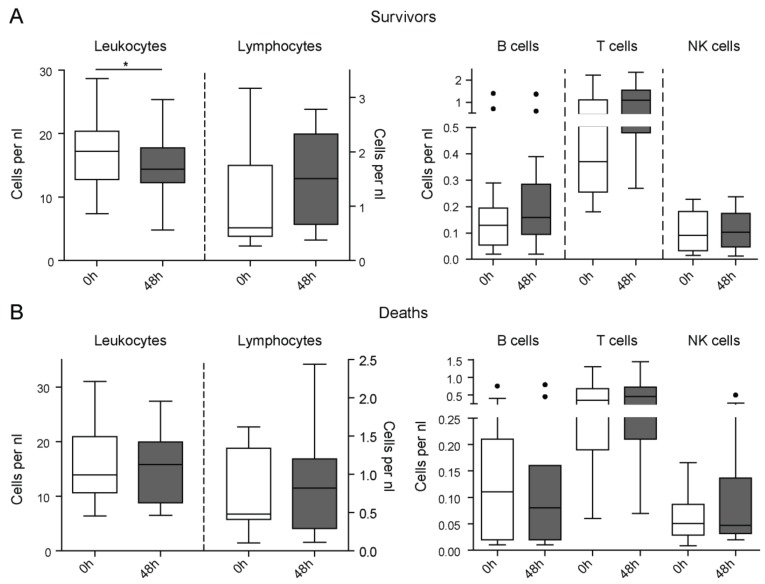
Longitudinal assessment of leukocyte subsets in ICU patients and prognosis. Peripheral blood leukocytes were assessed at ICU admission (0 h) as well as after 48 h (48 h) of ICU treatment in critically ill patients. (**a**) Leukocytes, lymphocytes, and their subsets per nanoliter in surviving ICU patients at admission and after 48 h of ICU treatment. (**b**) Leukocytes, lymphocytes, and their subsets per nanoliter in non-survivors at admission and after 48 h of ICU treatment. Statistics: * indicates *p* < 0.05. For comparison of two paired groups, the Wilcoxon signed rank test was used. Sample sizes with matched samples 0 h and 48 h: Deaths *n* = 11, survivors *n* = 17.

It had been hypothesized that the rapid restoration of innate and adaptive immune defects may promote a beneficial prognosis in patients with sepsis [4]. We were able to obtain follow-up samples in a subset of critically ill patients 48 h after admission to the ICU (*n* = 28). Strikingly, in patients that survived in the ICU (*n* = 17), we observed a reduction in leukocytes followed by a trend towards higher lymphocyte numbers at 48 h after ICU admission, that reached levels observed in healthy volunteers (Figure 4A). Moreover, T cell numbers increased in surviving ICU patients within 48 h, although the small number of patients with available follow-up samples did not allow to obtain significant results (Figure 4A). On the contrary, patients who died during the ICU treatment (*n* = 11) did not display such dynamics in their leukocytes or lymphocyte subpopulations (Figure 4B), supporting that the early restoration of immune cell alterations within 48 h in the ICU is a beneficial prognostic factor.

## 4. Discussion

It has been shown that the distribution of peripheral blood leukocyte subsets yields information about the severity and development of critical illness, especially sepsis [9]. In this study, we investigated the distributional changes of lymphocytes and their subsets in infected and septic patients using flow cytometry, which is a straightforward and reproducible method, on the basis of activation markers or ex vivo cytokine secretion. We found that numbers of T and NK cells were diminished in infected and in ICU patients compared to healthy controls, which was most likely due to the migration of those cells either to sites of infections and organ injury or to peripheral lymphatic tissue during the immune response [4,16]. Relative to each other, T cells were diminished, and B cells were increased in infection and sepsis, whereas NK cells did not change in frequency. Subsets of T and NK cells only showed minor shifts, i.e., a depletion of CD8^+^ T cells as well as a decline in CD16^+^ NK cells. These immunosuppressive features, in particular lymphopenia, have been previously linked to sepsis [7,8]. Interestingly, changes in B, T, and NK cell numbers were independent of sepsis in our study but represented a characteristic feature of critical illness. Our findings thereby indicate that the critical illness itself, rather than sepsis, may promote immunosuppression and consequently poor prognosis.

The rapid restoration of immune defects may be beneficial for the prognosis of patients with sepsis [4]. Our data fully support this concept, as patients that subsequently survived critical illness demonstrated significantly higher lymphocyte numbers at ICU admission, especially T and NK cells. As an essential part of the acquired immune response in infection, lymphocytes and their subsets play an important role in sepsis and survival. In a smaller study of 87 ICU patients with severe sepsis, the surviving patients showed higher counts of Th1 lymphocytes [17]. In protracted disease, several lymphocyte populations in patients with acute sepsis can upregulate the expression of receptors associated with cell exhaustion, such as CTLA4, TIM-3, LAG-3, or IL-7 receptor [6]. In our analysis, only T cells could predict the survival of the ICU patients, as low T cell counts were associated with a significantly increased risk of mortality. Our results are consistent with those of a large multicentric study [9]. The association between low number of circulating T cells and mortality may be due to different mechanisms. Firstly, the low number of circulating T cells may reflect a stronger migration of T cells into peripheral lymphatic tissue due to severe infection. Secondly, patients with an already low T cell count may have a reduce ability to counteract systemic inflammation and infections. Thirdly, reduced T cell numbers could be the result of an increased rate of cell death in critical illness, leading to compromised immune responses and thus increased risk of mortality. Undoubtedly, the overall reduced T cell counts are the net result of complex interactions, which may also include the induction of T cell lymphopenia by immature granulocytes [5].

It is important to openly mention the limitations of our study. We conducted a single-center study with a limited number of patients. While this approach allowed a high technical accuracy and reproducibility of the elaborate flow cytometric analysis, it restricts the options to conduct extensive subgroup analyses (e.g., based on age groups, etiology of disease, or site of infection). Possibly related to this, we identified several statistically significant correlations between laboratory parameters and lymphocytes and their subsets, but because of the weakness of those linear correlations, the clinical value must be considered low (or negligible) in all cases (Table 2). Furthermore, the follow-up of patients was limited. Larger prospective multicenter studies would very likely lead to more informative subgroup analyses and would allow for a better definition of the predictive value of T cells for the survival of critically ill patients.

Although only a subgroup of our ICU patient cohort was available for longitudinal follow-up analysis, our study revealed a clear trend of a rapid restoration of lymphopenia and low T cell counts within 48 h in patients that subsequently survived the critical illness, while patients that subsequently died failed to restore these alterations within 48 h. Our data thereby support the concept of therapeutically augmenting immune responses in critical illness by novel, tailored therapeutic interventions in intensive care medicine [10], for example the restoration of T cell exhaustion and of T cell function in sepsis [6]. As supported by our study, altered immune cell numbers, composition, and function not only determine responses to infectious threats but also appear to be decisive for the course of critical illness and organ failure per se. Future studies should aim at validating our findings in larger longitudinal sampling and may determine the optimal strategy and timing for such novel therapeutic interventions in critical care medicine.

## 5. Conclusions

We conclude, that our flow-cytometric analysis of peripheral blood revealed striking changes in circulating lymphocyte subsets in critically ill patients, namely depletion of lymphocytes, T cells and NK cells in infected and in ICU patients, which was independent of sepsis. Furthermore, lymphopenia and depletion of T cells at ICU admission were linked to increased mortality, supporting their relevance as predictive biomarkers and potential therapeutic targets in critically ill patients.

## Figures and Tables

**Table 1:** Characteristics of healthy volunteers, standard care patients with bacterial infections, and intensive care patients.

Parameter	HC	SC	ICU	ICU: No Sepsis	ICU: Sepsis
Number *n*	63	50	105	53	52
Sex (male/female) *n*	37/26	33/17	66/39	35/18	31/21
Age (years)	48 (22–77)	66.5 (21–90)	66 (18–97)	60 (23–92)	68 (18–97)
RBC (per nL)	4.8 (4.2–6.7)	4.15 (2.2–6.2)	3.5 (2–9.4)	3.7 (2–6.3)	3.4 (2.1–9.4)
Hemoglobin (g/dL)	14.1 (12–16)	12.5 (7.1–17.5)	10.2 (5.6–16.1)	10.8 (5.6–15.5)	9.55 (6.2–16.1)
Hematocrit (%)	41.4 (36.1–46.1)	36.9 (23.7–51.3)	30.9 (16.6–50.5)	32 (16.6–50.5)	29.8 (19.1–50.3)
WBC (per nL)	5.8 (3.7–10)	9.2 (1.7–23)	13.5 (0.5–42.9)	10.6 (2.7–31.4)	15.4 (0.5–42.9)
Lymphocytes (per nL)	1.86 (0.72–3.13)	0.75 (0–3.46)	0.64 (0–6.86)	0.75 (0–3.46)	0.53 (0.02–6.86)
B cells (per nL)	0.13 (0.04–0.60)	0.066 (0–0.55)	0.088 (0–1.40)	0.10 (0–0.66)	0.064 (0–1.40)
T cells (per nL)	1.34 (0.27–2.54)	0.66 (0.09–2.67)	0.43 (0–3.28)	0.51 (0–2.09)	0.34 (0.01–3.28)
NK cells (per nL)	0.26 (0.08–0.78)	0.11 (0.01–0.79)	0.084 (0–2.59)	0.10 (0–1.65)	0.053 (0–2.59)

The median and range are given, unless indicated otherwise. The two right columns differentiate the characteristics of ICU patients with or without sepsis. Abbreviations: WBC: white blood cell count, RBC: red blood cell count, HC: healthy control, SC: standard care patients, ICU: intensive care unit; NK cells: natural killer cells.

**Table 2:** Correlation between leukocytes, lymphocytes, and their subsets with laboratory parameters of ICU patients.

	Leukocytes		Lymphocytes		B Cells		T Cells		NK Cells	
	*r*	*p*-Value	*r*	*p*-Value	*r*	*p*-Value	*r*	*p*-Value	*r*	*p*-Value
CRP	0.215	0.030	−0.133	n.s.	−0.176	n.s.	−0.090	n.s.	−0.153	n.s.
PCT	0.064	n.s.	−0.236	0.025	−0.136	n.s.	−0.146	n.s.	−0.444	0.000
IL10	0.162	n.s.	−0.316	0.003	−0.123	n.s.	−0.305	0.004	−0.223	0.037
CX3CL1	−0.091	n.s.	−0.062	n.s.	−0.034	n.s.	−0.024	n.s.	−0.271	0.016
GFR	−0.204	0.038	−0.016	n.s.	−0.154	n.s.	0.002	n.s.	0.092	n.s.
SCr	0.197	0.045	0.023	n.s.	0.137	n.s.	0.020	n.s.	−0.108	n.s.

Abbreviations: CRP: C-reactive protein, PCT: procalcitonin, IL10: interleukin-10, CX3CL1: fractalkine, GFR: glomerular filtration rate, SCr: serum creatinine, r: Spearman’s rho correlation coefficient, *p* value: significance level; n.s.: not significant.
