# Erratum: Hohlstein, P. et al. Prognostic Relevance of Altered Lymphocyte Subpopulations in Critical Illness and Sepsis. *Journal of Clinical Medicine* 2019, *8*, 353

**DOI:** 10.3390/jcm8091296

**Published:** 2019-08-23

**Authors:** Philipp Hohlstein, Hendrik Gussen, Matthias Bartneck, Klaudia Theresa Warzecha, Christoph Roderburg, Lukas Buendgens, Christian Trautwein, Alexander Koch, Frank Tacke

**Affiliations:** 1 Department of Medicine III, RWTH-University Hospital Aachen, 52074 Aachen, Germany; 2 Department of Gastroenterology/Hepatology, Charité University Medical Center Berlin, 13353 Berlin, Germany

The authors wish to make the following corrections to this paper [1].

The authors would like to clarify the Author Contributions section in order to avoid its being unintentionally vague and potentially confusing. Accordingly,

**Author Contributions:** P.H. and H.G. collected blood samples, performed FACS analyses, and conducted data analyses. M.B. and K.T.W. supported the flow-cytometric experiments. C.R., L.B., and C.T. contributed to data collection and analysis. A.K. and F.T. designed and supervised the study. P.H. and F.T. wrote the manuscript. All authors corrected and approved the manuscript.

should be replaced with

**Author Contributions:** P.H. and H.G. collected blood samples, performed FACS analyses, and conducted data analyses. M.B. and K.T.W. supported the flow-cytometric experiments. C.R., L.B., and C.T. contributed to data collection and analysis. A.K. and F.T. designed and supervised the study. P.H. drafted the manuscript and F.T. corrected the manuscript. All authors corrected and approved the manuscript.

The authors apologize for any inconvenience that these changes may have caused to the readers.
